# Functional Roles of the N- and C-Terminal Regions of the Human Mitochondrial Single-Stranded DNA-Binding Protein

**DOI:** 10.1371/journal.pone.0015379

**Published:** 2010-10-28

**Authors:** Marcos T. Oliveira, Laurie S. Kaguni

**Affiliations:** Department of Biochemistry and Molecular Biology, Center for Mitochondrial Science and Medicine, and Graduate Program in Genetics, Michigan State University, East Lansing, Michigan, United States of America; University of Medicine and Dentistry of New Jersey, United States of America

## Abstract

Biochemical studies of the mitochondrial DNA (mtDNA) replisome demonstrate that the mtDNA polymerase and the mtDNA helicase are stimulated by the mitochondrial single-stranded DNA-binding protein (mtSSB). Unlike *Escherichia coli* SSB, bacteriophage T7 gp2.5 and bacteriophage T4 gp32, mtSSBs lack a long, negatively charged C-terminal tail. Furthermore, additional residues at the N-terminus (notwithstanding the mitochondrial presequence) are present in the sequence of species across the animal kingdom. We sought to analyze the functional importance of the N- and C-terminal regions of the human mtSSB in the context of mtDNA replication. We produced the mature wild-type human mtSSB and three terminal deletion variants, and examined their physical and biochemical properties. We demonstrate that the recombinant proteins adopt a tetrameric form, and bind single-stranded DNA with similar affinities. They also stimulate similarly the DNA unwinding activity of the human mtDNA helicase (up to 8-fold). Notably, we find that unlike the high level of stimulation that we observed previously in the *Drosophila* system, stimulation of DNA synthesis catalyzed by human mtDNA polymerase is only moderate, and occurs over a narrow range of salt concentrations. Interestingly, each of the deletion variants of human mtSSB stimulates DNA synthesis at a higher level than the wild-type protein, indicating that the termini modulate negatively functional interactions with the mitochondrial replicase. We discuss our findings in the context of species-specific components of the mtDNA replisome, and in comparison with various prokaryotic DNA replication machineries.

## Introduction

**Figure 1:**
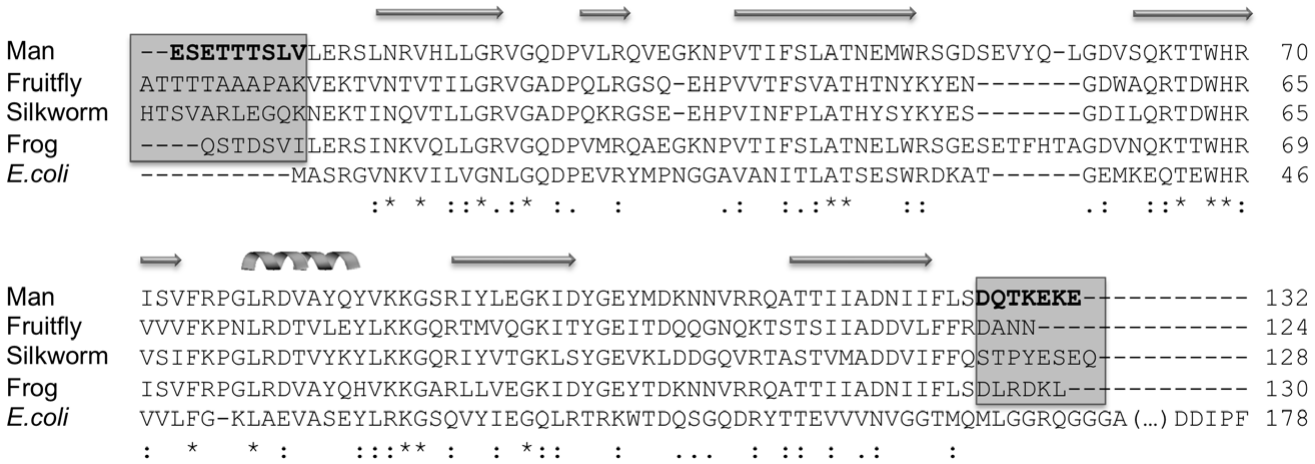
Sequence alignment of animal mtSSBs with *E. coli* SSB, and mutagenesis of *Hs*mtSSB. Thirteen mtSSB sequences (notwithstanding the mitochondrial presequence) from various species across the animal kingdom were aligned with the *E. coli* SSB sequence using Clustal X [49]. Only the representative mtSSB sequences from humans (GenBank accession: NP_003134), fruitfly (*Drosophila melanogaster*; GenBank accession: AAF16936), silkworm (*Bombyx mori*; GenBank accession: ABF51293), and frog (*Xenopus laevis*; GenBank accession: NP_001095241) are shown. The gray boxes denote the N- and C-terminal regions of animal mtSSB that are not conserved in *E. coli* SSB. The amino acid residues indicated in bold in the *Hs*mtSSB sequence were targeted for deletion mutagenesis. Amino acid residues 13–123 in the *Hs*mtSSB sequence comprise the OB-fold domain (see the text for details).

Single-stranded DNA-binding proteins (SSBs) are essential components in DNA metabolic processes, including replication, repair and recombination. In addition to their protective single-stranded DNA-coating properties, it has been demonstrated that SSBs from distantly-related species have far more complex roles that include the organization and/or mobilization of all aspects of DNA metabolism (reviewed in [1]). In eukaryotic cells, there are two compartmentalized SSBs: replication protein A is found in the nucleus, whereas mtSSB is found in the mitochondrion. Despite sharing a similar single-stranded DNA-binding domain (oligonucleotide/oligosaccharide binding domain or OB-fold) together with bacterial and viral SSBs [2], [3], [4], [5], [6], and performing analogous functions in their respective cellular compartments, mtSSB and RPA are not related evolutionarily. mtSSBs are homologues of eubacterial SSBs, whose prototype is the well studied *Escherichia coli* SSB (*Ec*SSB) (Fig. 1), a fact that is in agreement with the endosymbiont theory of mitochondrial origin [7].

At the mitochondrial DNA (mtDNA) replication fork, mtSSB interacts functionally with DNA polymerase γ (pol γ) and mtDNA helicase (also known as Twinkle) to promote mtDNA replication. *In vitro*, the *Drosophila melanogaster* mtSSB (*Dm*mtSSB) stimulates 15- to 20-fold the DNA polymerase and 3′-5′ exonuclease activities of *Drosophila* pol γ (*Dm*pol γ) on a singly-primed single-stranded DNA (ssDNA) template [8]. The human mtSSB (*Hs*mtSSB) stimulates the DNA unwinding activity of the human mtDNA helicase (*Hs*mtDNA helicase) [9], and is required for strand-displacement DNA synthesis in the presence of human pol γ (*Hs*pol γ) and *Hs*mtDNA helicase [10]. *In vivo*, deleterious mutations in the mtSSB gene cause loss of mtDNA that results in developmental arrest in *Drosophila* [11], and growth limitation due to mitochondrial dysfunction in *Saccharomyces cerevisiae* [12]. Our group has shown that the knockdown of the endogenous *Dm*mtSSB in *Drosophila* Schneider cells results in mtDNA copy number reduction and growth retardation [13]. The overexpression of the wild-type protein rescues the phenotype, whereas a ssDNA-binding mutant of *Dm*mtSSB is unable to do so. Very recently, the knockdown of *Hs*mtSSB in human HeLa cells was shown to promote a gradual decline in mtDNA copy number and a severe reduction in 7S DNA synthesis [14].

**Figure 2:**
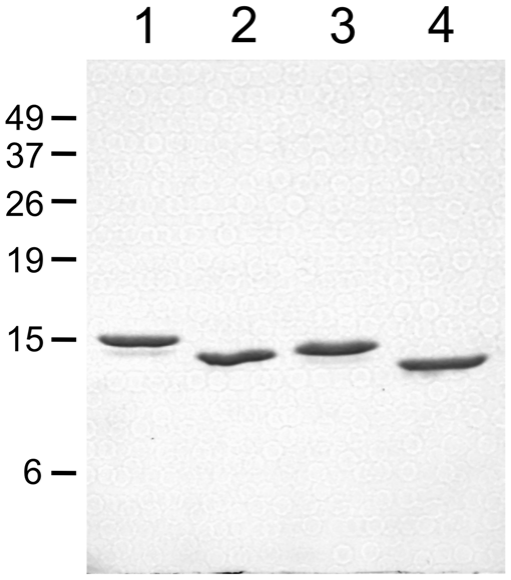
SDS-PAGE of terminal deletion variants of *Hs*mtSSB. Near-homogeneous fractions (∼2 µg) of recombinant *Hs*mtSSBwt (*lane 1*), ΔN (*lane 2*), ΔC (*lane 3*), and ΔNΔC (*lane 4*) were subjected to SDS-PAGE in a 17% gel, followed by Coomassie blue staining as described under “Materials and Methods.” The sizes of molecular mass markers (BenchMark™ Pre-Stained Protein Ladder, Invitrogen™) are indicated in kDa at *left*.

In this report, we examine the biochemical and physical properties of four *Hs*mtSSB proteins: the mature full-length protein (notwithstanding the mitochondrial presequence, *Hs*mtSSBwt), a deletion variant lacking the first 9 residues in the N-terminus (*Hs*mtSSBΔN), a deletion variant lacking the last 7 residues in the C-terminus (*Hs*mtSSBΔC), and a variant that lacks both termini (*Hs*mtSSBΔNΔC). The target regions are of particular interest for several reasons: 1) they represent two of the few regions of significant amino acid sequence variability between mtSSBs and bacterial SSBs – the N-terminal extension is absent in bacterial SSBs, whereas the C-terminus is short and uncharged in mtSSBs (Fig. 1); 2) they appear disordered in the crystal structure of *Hs*mtSSB [3], suggesting flexibility and/or dynamism of these regions without apparent interactions with the ssDNA-binding domain; and 3) in *Ec*SSB, bacteriophage T7 gp2.5 and T4 gp32 SSBs, the C-terminal region interacts with other components of DNA metabolic processes, and regulates ssDNA binding negatively. We evaluate our findings in the context of functions at the mitochondrial replication fork, and discuss them in comparison with other DNA replication systems.

## Results

### Purification of amino- and carboxyl-terminal deletion variants of *Hs*mtSSB, and determination of oligomeric state

In order to study the possible roles of the N- and C-terminal regions of *Hs*mtSSB at the mtDNA replication fork, we produced three deletion variants together with *Hs*mtSSBwt: *Hs*mtSSBΔN, *Hs*mtSSBΔC, and *Hs*mtSSBΔNΔC. The overexpression of untagged recombinant proteins in *E. coli* using the pET-11a system resulted in high levels of soluble proteins, except for *Hs*mtSSBΔC; the extraction and solubility of *Hs*mtSSBΔC was dependent on inclusion of a dodecyl-maltoside detergent. From this point on, the purification of the proteins was identical. We modified earlier protocols for purification of *Dm*mtSSB [8] and *Hs*mtSSB [15] in order to obtain highly pure proteins (Fig. 2). In particular, substitution of velocity sedimentation, used previously as a final step, by phosphocellulose chromatography followed by assay of ATP-dependent dsDNA unwinding activity in each fraction, was a critical improvement towards the elimination of a highly active bacterial contaminant (see “Materials and Methods” for details).

**Figure 3:**
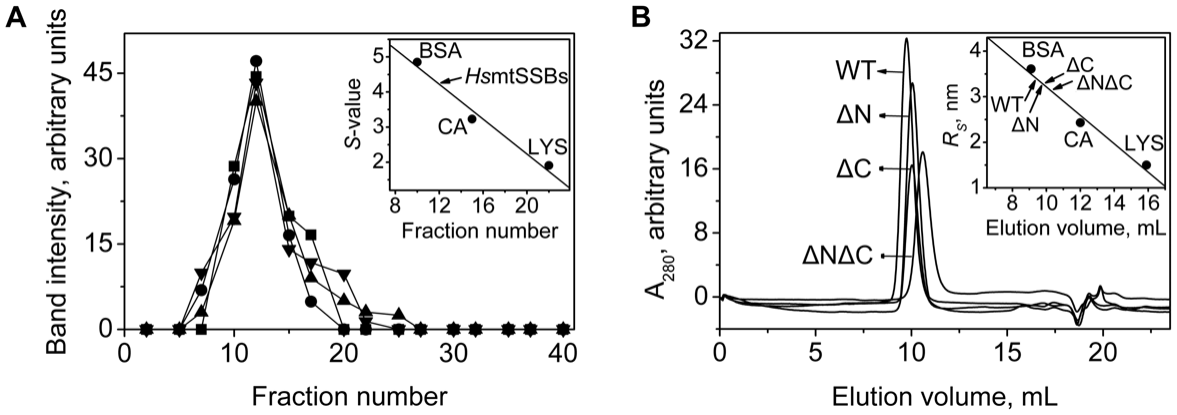
Terminal deletion variants of *Hs*mtSSB form tetramers in solution. Hydrodynamic analysis of *Hs*mtSSBwt and deletion variants. **A**, *Hs*mtSSBwt (•), ΔN (▪), ΔC (▴), and ΔNΔC (▾) were sedimented in 12–30% glycerol gradients for 63 hr at 264,000×*g*, and the gradient fractions were analyzed by 17% SDS-PAGE. **B**, the *Hs*mtSSB proteins were chromatographed on a Superdex 75 gel filtration column, and fractions were analyzed by absorption at 280 nm. Standard protein markers used were: bovine serum albumin (BSA: 4.85 *S*, *R_S_* 3.61 nm), carbonic anhydrase (CA: 3.23 *S*, *R_S_* 2.43 nm), and lysozyme (LYS: 1.91 *S*, *R_S_* 1.5 nm).

With near-homogeneous *Hs*mtSSBwt and the terminal deletion variants in hand, we evaluated the possible consequences of the lack of the termini on its physical and biochemical properties. SDS-PAGE of *Hs*mtSSBwt, ΔN, ΔC, and ΔNΔC reveals polypeptides of ∼15, 14, 14, and 13 kDa, respectively (Fig.2). To investigate their oligomeric state in solution, we employed hydrodynamic methods to estimate native molecular mass. We observed single peaks for each recombinant protein both in velocity sedimentation and in Superdex 75 gel filtration (Fig. 3). The sedimentation coefficient was 4.2 *S* for all of the proteins, and the Stokes' radii were 3.4, 3.2, 3.2, and 3.0 nm respectively for wild type, ΔN, ΔC, and ΔNΔC. Together, these indexes indicate native molecular masses of ∼56, 53, 53, and 51 kDa, respectively, consistent with the size of homotetrameric forms.

### Terminal deletion variants of *Hs*mtSSB bind to ssDNA with similar affinities

**Figure 4:**
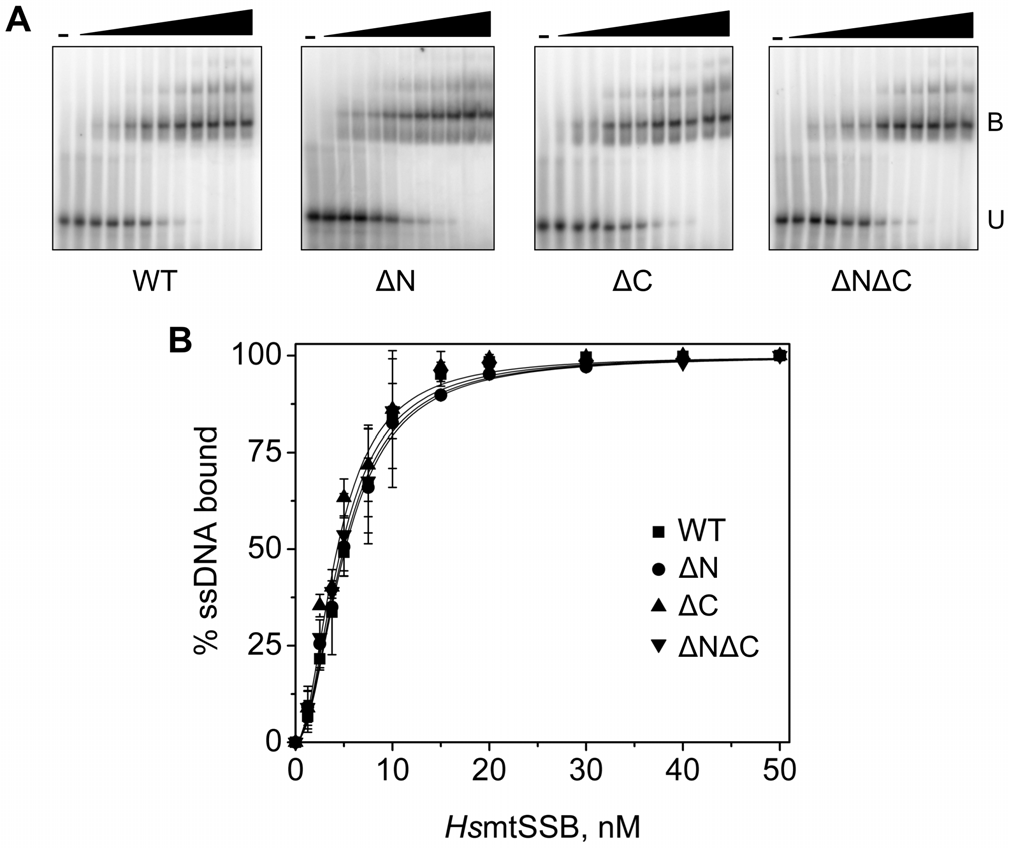
Terminal deletion variants of *Hs*mtSSB bind to ssDNA with similar affinities. **A**, ssDNA-binding affinity was evaluated by GMSA. *Hs*mtSSBwt and its deletion variants were pre-incubated with a radiolabeled 48-mer oligonucleotide at 50 mM NaCl in the presence of increasing mtSSB concentrations: 1.25, 2.5, 3.75, 5, 7.5, 10, 15, 20, 30, 40 and 50 nM (as tetramer), as described under “Materials and Methods”. “−” denotes no added protein. The fraction of unbound (U) and bound (B) oligomer were quantitated, and the data were plotted in **B** as the percent of substrate utilized. The data represent the average of three experiments.

We proceeded to evaluate the *Hs*mtSSB deletion variants by examining their biochemical activities as compared to the wild-type protein. Using a gel mobility shift assay (GMSA), we examined the apparent DNA-binding affinities of *Hs*mtSSBwt and the terminal deletion variants using a 48-mer ssDNA oligonucleotide (Fig. 4), whose size is close to the binding-site size determined previously for *Hs*mtSSB [16]. A titration of the proteins at 50 mM NaCl revealed no significant differences in ssDNA-binding affinities between *Hs*mtSSBwt and the deletion variants, with apparent *K_d_*s of ∼5 nM. Interestingly, the lack of the C-terminal region does not appear to interfere with the ssDNA-binding efficacy of *Hs*mtSSB, in contrast to *Ec*SSB and T7 gp2.5. A recent report by Kozlov *et al.* [17] shows that an increased ssDNA binding of *Ec*SSB lacking the C-terminus is observed only at 100 and 200 mM NaCl, and not at low salt concentration (20 mM NaCl). We also performed the GMSA assays with the *Hs*mtSSB proteins at 20 and 100 mM NaCl, but failed to observe any differences in binding affinities (data not shown). At the 20–100 mM range, salt does not seem to affect the ssDNA-binding affinity of either the wild-type or variant forms of *Hs*mtSSB.

### Salt-dependent stimulation of *Hs*pol γ by *Hs*mtSSB terminal deletion variants

**Figure 5:**
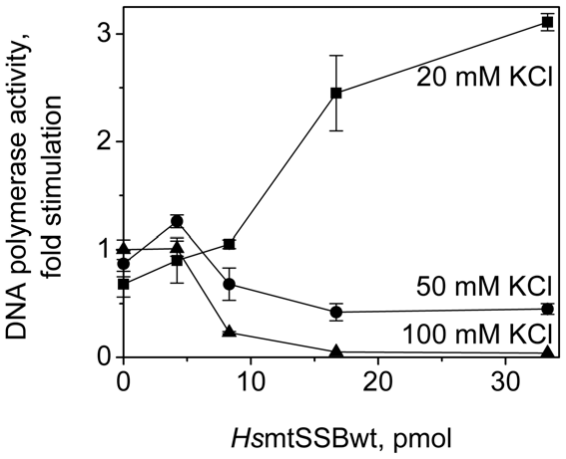
*Hs*mtSSBwt stimulates the DNA polymerase activity of *Hs*pol γ in a salt-dependent manner. DNA synthesis by reconstituted *Hs*pol γ holoenzyme was measured on singly-primed M13 DNA, as described under “Materials and Methods”, in the presence of the indicated KCl and *Hs*mtSSBwt concentrations. The data were normalized to the amount of nucleotide incorporated by *Hs*pol γ at 100 mM KCl in absence of *Hs*mtSSBwt (that was arbitrarily set as 1).

To evaluate the capacity of the *Hs*mtSSB variants to stimulate the DNA polymerase activity of *Hs*pol γ *in vitro*, we first examined the effects of KCl concentration on DNA synthesis by reconstituted *Hs*pol γ holoenzyme on singly-primed M13 DNA in the presence and absence of *Hs*mtSSBwt (Fig. 5). In the absence of *Hs*mtSSBwt, *Hs*pol γ activity is stimulated ∼2 fold as the concentration of KCl in the reaction increases from 20 to 100 mM, in agreement with previously published data [18]. Unlike *Dm*pol γ stimulation by *Dm*mtSSB that occurs over a broad range of KCl concentrations (0–130 mM) [8], the stimulation of *Hs*pol γ by *Hs*mtSSBwt is only observed at 20 mM KCl, and reaches its maximum at a concentration of *Hs*mtSSBwt sufficient to cover the entire singly-primed M13 DNA substrate (according to our GMSA data). At 50 mM KCl, the presence of *Hs*mtSSBwt at low levels promotes a slight stimulation of DNA synthesis by *Hs*pol γ, but it becomes somewhat inhibitory at higher levels. At 100 mM KCl, where the activity of *Hs*pol γ alone is highest, the presence of *Hs*mtSSBwt is completely inhibitory. The maximal stimulation of DNA synthesis by *Hs*pol γ in the presence of *Hs*mtSSBwt at 20 mM KCl is only moderate, albeit ∼3-fold higher than the activity of *Hs*pol γ alone at 100 mM KCl. Judging by the fact that the ssDNA-binding affinity of *Hs*mtSSBwt does not change over the range of 20–100 mM KCl, the data suggest that increasing ionic strength inhibits the ability of *Hs*pol γ to displace *Hs*mtSSB from ssDNA template during the course of *in vitro* DNA synthesis.

**Figure 6:**
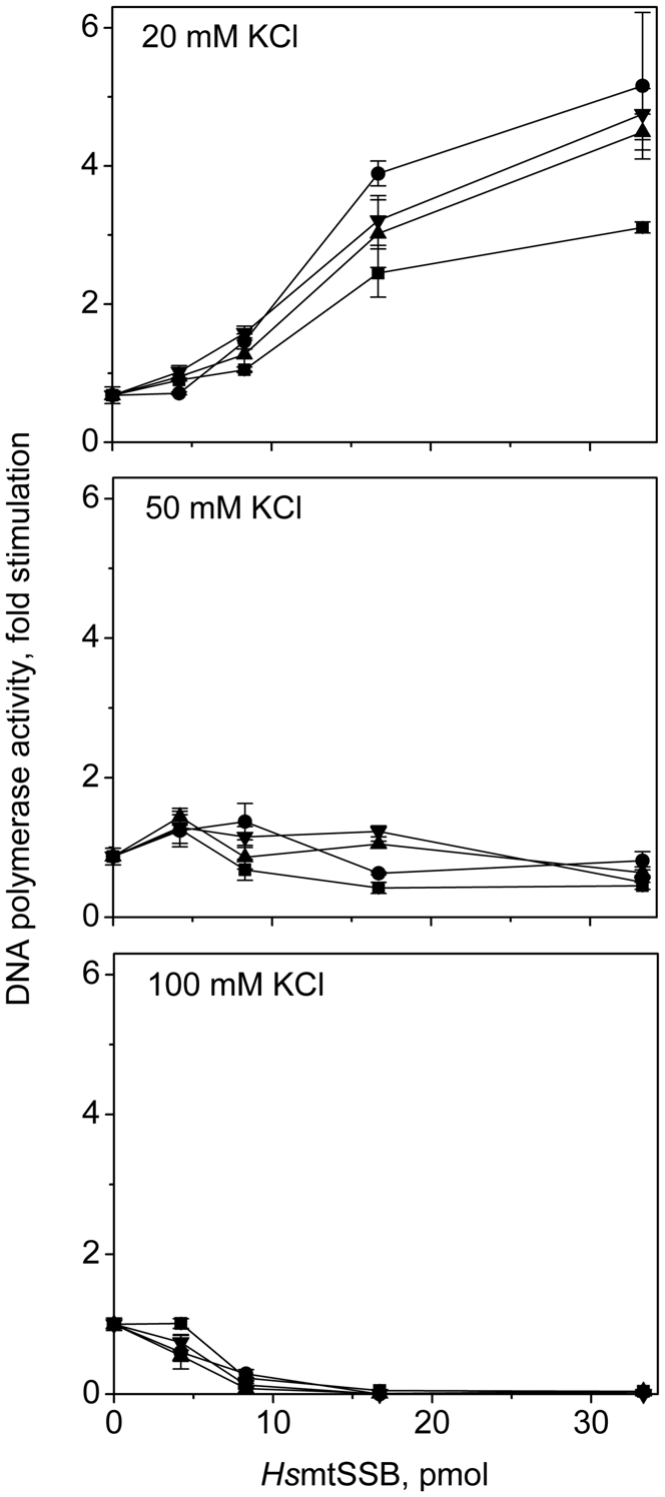
Stimulation of *Hs*pol γ by terminal deletion variants of *Hs*mtSSB. DNA synthesis by reconstituted *Hs*pol γ holoenzyme was measured on singly-primed M13 DNA, as described under “Materials and Methods”, in the presence of the indicated concentrations of *Hs*mtSSBwt (▪), ΔN (•), ΔC (▴), and ΔNΔC (▾), at 20 mM (upper), 50 mM (middle), and 100 mM (lower panel) KCl. The data were normalized to the amount of nucleotide incorporated by *Hs*pol γ at 100 mM KCl in absence of *Hs*mtSSBwt (that was arbitrarily set as 1).

Next, we evaluated the ability of the terminal deletion variants of *Hs*mtSSB to stimulate DNA synthesis by *Hs*pol γ (Fig. 6). Overall, the effect of KCl concentration is similar among the variants: at 20 mM, DNA synthesis is stimulated; at 50 mM, we observe no stimulation and/or slight inhibition; at 100 mM, *Hs*pol γ is inhibited completely. Notably, under low salt conditions, *Hs*mtSSBΔN, ΔC, and ΔNΔC show 1.4- to 2-fold higher stimulation of *Hs*pol γ as compared to *Hs*mtSSBwt. This increased stimulation is not apparent at low concentrations of the *Hs*mtSSBs, but it is clearly evident and reproducible at saturating levels. The data argue that the N- and C-terminal regions of *Hs*mtSSB have functionally inhibitory roles on its ability to stimulate the DNA polymerase activity of *Hs*pol γ, suggesting a modulatory role *in vivo*.

### Stimulation of DNA unwinding activity of *Hs*mtDNA helicase by terminal deletion variants of *Hs*mtSSB

**Figure 7:**
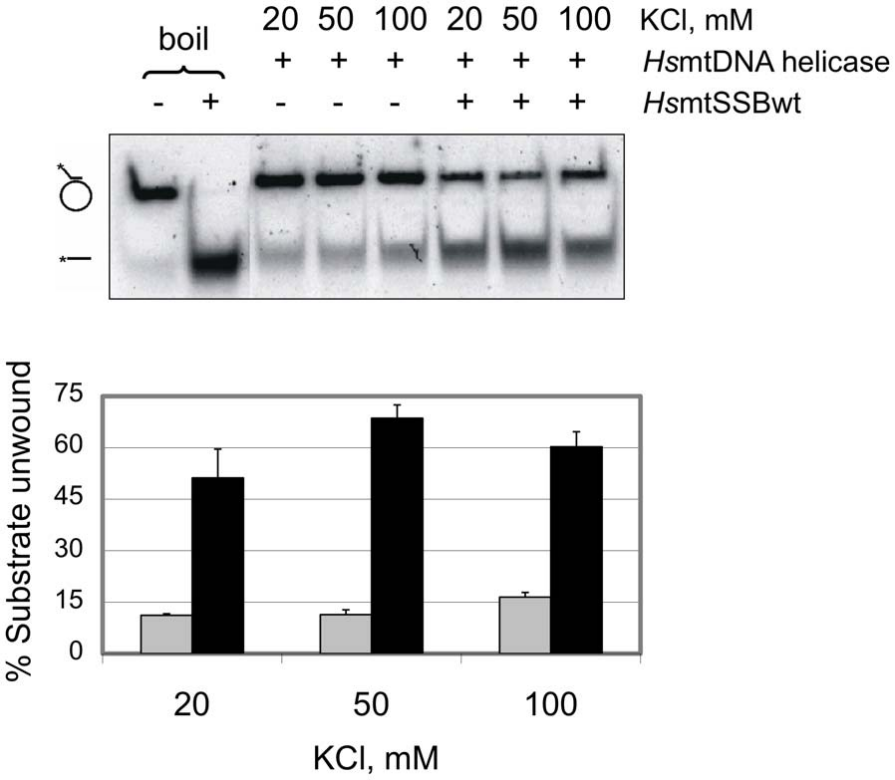
Stimulation of DNA unwinding activity of *Hs*mtDNA helicase by *Hs*mtSSBwt is independent of KCl concentration. DNA unwinding assays were performed as described under “Materials and Methods”. Upper panel, 3.5 nM of *Hs*mtDNA helicase (as hexamer) and 200 nM of *Hs*mtSSBwt (as tetramer) were used where indicated by “+”. The concentration of KCl used is indicated above each lane. “−” and “+ boil” lanes represent the intact and denatured substrate (heated to 100°C for 2 min prior to loading), respectively. Lower panel, analysis of the data shown in the upper panel together with the data from two other independent experiments. The gray and black bars represent the average of unwound substrate as percent in the absence and presence of *Hs*mtSSBwt, respectively.

As shown previously, *Hs*mtSSBwt stimulates the DNA unwinding activity of *Hs*mtDNA helicase *in vitro* [9]. We examined the effects of the terminal deletion variants in stimulating the DNA unwinding activity of *Hs*mtDNA helicase using as substrate pBSKS+ ssDNA (2,958 nt), to which was annealed a 60-mer ssDNA oligonucleotide that creates a 40-nt 5′-single-stranded tail for helicase loading followed by 20 nt of paired sequence. First, we asked if varying KCl concentrations produced the same pattern of stimulation of *Hs*mtDNA helicase by *Hs*mtSSBwt as compared to its stimulation of *Hs*pol γ (Fig. 7). We found that *Hs*mtDNA helicase shows a slightly better DNA unwinding activity at 100 mM KCl as compared to that at 20 and 50 mM KCl. We then evaluated various potassium salts, including phosphate, acetate and glutamate, and found that KCl provided the best stimulation (data not shown). In contrast to the results with *Hs*pol γ, stimulation of *Hs*mtDNA helicase in the presence of saturating amounts of *Hs*mtSSBwt is similar at the three KCl concentrations tested, suggesting that the results we show in Figures 5 and 6 are related specifically to functional interactions between *Hs*mtSSB and *Hs*pol γ.

**Figure 8:**
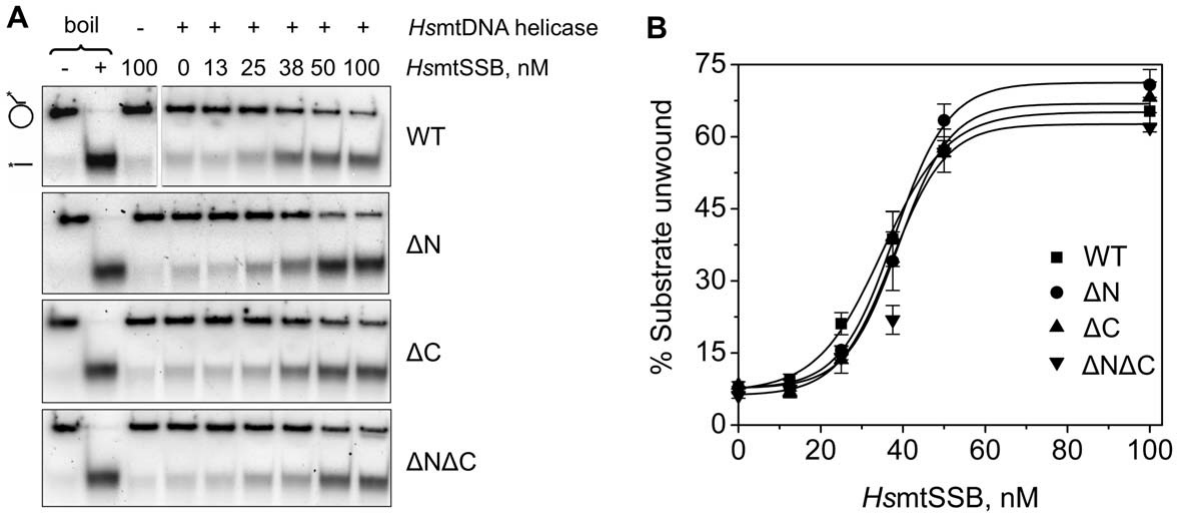
Stimulation of *Hs*mtDNA helicase by terminal deletion variants of *Hs*mtSSB. DNA unwinding assays were performed as described under “Materials and Methods”. **A**, a constant concentration of *Hs*mtDNA helicase (3.5 nM as hexamer) and KCl (50 mM) were used in each assay. The concentration of *Hs*mtSSBs (as tetramer) that was used is indicated above each lane. “−” and “+ boil” lanes represent the intact and denatured substrate (heated to 100°C for 2 min prior to loading), respectively. **B**, analysis of the data shown in **A** together with the data from two other independent experiments. The data represent the average of unwound substrate as percent.

We extended our analysis by titrating *Hs*mtSSBwt, ΔN, ΔC, and ΔNΔC in DNA unwinding assays conducted at 50 mM KCl (Fig. 8). None of the concentrations of the *Hs*mtSSBs used were sufficient to cause any dsDNA destabilization in the absence of helicase (Fig. 8A). *Hs*mtDNA helicase shows maximal DNA unwinding activity in the presence of 100 nM *Hs*mtSSB, a concentration corresponding to coating of ∼80% of the ssDNA substrate (according to our GMSA data). No significant differences in stimulation were observed between *Hs*mtSSBwt and deletion variants; stimulation of the *Hs*mtDNA helicase was ∼8 fold at the highest *Hs*mtSSB concentrations.

## Discussion

Biochemical studies of the mtDNA replisome demonstrate that pol γ and the mtDNA helicase are stimulated by mtSSB. We have shown previously that *Dm*mtSSB can stimulate ∼20-fold *in vitro* DNA synthesis by *Dm*pol γ over a broad range of KCl concentrations, primarily by enhancing primer recognition and binding [8], [19]. Similarly, *Dm*mtSSB stimulates ∼15-fold the 3′-5′ exonuclease activity of *Dm*pol γ over the same KCl range [8]. Furthermore, *Hs*mtSSB has been shown to stimulate the DNA unwinding activity of the *Hs*mtDNA helicase [9] and together with *Hs*pol γ and *Hs*mtDNA helicase, reconstitutes a minimal mtDNA replisome *in vitro* [10]. These data indicate that mtSSBs serve an important role in initiation and elongation of DNA synthesis in mtDNA replication, consistent with the observation that the disruption of the *Drosophila* (*lopo*) and yeast (*rim1*) mtSSB genes results in loss of mtDNA and respiratory capacity, and consequently, developmental lethality or impaired growth, respectively [11], [12].

mtSSBs are homologues of *Ec*SSB, with which they exhibit a high degree of amino acid sequence conservation in the OB-fold domain (Fig. 1), and share similar physical, biochemical, and structural properties [3], [16], [19], [20]. Animal mtSSBs, however, evolved at least two different sequences from their eubacterial counterpart: their N- and C-termini. After import into mitochondria, the mitochondrial presequence is cleaved, producing the mature mtSSB protein. The mature polypeptide in humans contains 9 amino acids at the N-terminus that are absent in *Ec*SSB, and only 7 residues at the C-terminus, in contrast with the long acidic C-terminal tail of its eubacterial homologue; these potentially protrude from the ssDNA-binding core without any detectable secondary structure [3]. Here, we sought to analyze the functional importance of the N- and C-terminal regions of *Hs*mtSSB in the context of mtDNA replication. We purified *Hs*mtSSBwt, *Hs*mtSSBΔN, *Hs*mtSSBΔC, and *Hs*mtSSBΔNΔC to near-homogeneity and showed that all of the proteins form tetramers in solution, indicating that the termini are not critical for the folding or stability of *Hs*mtSSB, as predicted by the crystallographic data [3].

Next, we analyzed the ssDNA-binding properties of the terminal deletion variants using a GMSA approach. The lack of either or both termini did not alter the ssDNA-binding affinity of *Hs*mtSSB, which is relatively high as observed for SSBs from various sources [21]. This is particularly relevant because we have shown previously that ssDNA-binding variants of *Dm*mtSSB fail to stimulate *Dm*pol γ efficiently *in vitro*, and promote mtDNA depletion and cell growth defects in *Drosophila* Schneider cells [13]. Our findings distinguish biochemically the role of the C-terminal region of mtSSBs as compared to prokaryotic forms. The crystal structures of viral, eubacterial, eukaryotic nuclear and mitochondrial SSBs show that these proteins share a common structural domain for binding to ssDNA, the OB-fold [2], [3], [4], [5], [6], although they share no sequence homology. In addition, eubacterial and bacteriophage SSBs do share another structural feature: a long acidic C-terminal tail, which is essential for DNA replication and viability of the organisms [22], [23], [24]. The removal of the C-termini of *Ec*SSB, T7 gp2.5 and T4 gp32 increases their ssDNA-binding affinities significantly [22], [24], [25] but at the same time, abolishes interactions with other components of their cognate replication machinery [1], [26], [27], [28]. Recently, Shereda *et al.* [29] showed that proteins that interact with the C-terminus of *E. coli* SSB share a similar structural surface where the interaction occurs, demonstrating the presence of a signature contact structure. Interaction with the C-terminus of *E. coli* SSB via this signature structure appears to be highly regulated, because progressive truncations from its C-terminal end cause a progressive loss of both physical and functional interactions. Marintcheva *et al.* [30] showed that the acidic C-terminus of T7 gp2.5 and ssDNA actually compete for binding to the ssDNA-binding cleft of the protein, which is located in the N-terminal OB-fold domain. A functional model proposes that in absence of DNA, the C-terminal region binds to the ssDNA-binding cleft, and is then displaced upon ssDNA binding, rendering it available for protein-protein interactions. Such an interaction between the C-terminus and the ssDNA-binding cleft is suggested to create an electrostatic shield that protects the binding cleft from random charged surfaces inside the cell. Our data shows clearly that the C-terminal region of *Hs*mtSSB does not influence the ssDNA-binding affinity of the protein, suggesting that this region of the eubacterial-like mtSSBs serves a role that differs from those of *Ec*SSB, T7 gp2.5 and T4 gp32. Indeed, in this sense, *Hs*mtSSB resembles the nuclear replication protein A (reviewed in [31]); binding of random charged molecules to the ssDNA-binding cleft of eukaryotic SSBs is thus most likely prevented by a distinct mechanism.

To investigate further the functional properties of the N- and C-terminal regions of *Hs*mtSSB, we performed stimulation assays of *Hs*pol γ and *Hs*mtDNA helicase under varying conditions. We found that the stimulation of the DNA polymerase activity of *Hs*pol γ by *Hs*mtSSBwt is moderate (∼3 fold) and observed only at low KCl concentrations (20 mM). Increasing KCl concentrations resulted in concentration-dependent inhibition, suggesting that electrostatic forces govern the functional interactions between *Hs*pol γ and *Hs*mtSSB. Interestingly, *in vitro* stimulation (up to 20 fold) of the DNA polymerase activity of *Dm*pol γ by *Dm*mtSSB is observed over a broad range of KCl concentrations (0 to 130 mM) [8]. We speculate that this difference in the human and *Drosophila* systems likely reflects the different subunit composition of the mammalian and insect pol γs. *Hs*pol γ is a heterotrimeric enzyme comprising one catalytic and two accessory subunits (αβ_2_) [32], [33]; the α and dimeric β subunits can be expressed in heterologous systems and purified independently [15], [18], and the holoenzyme is subsequently reconstituted *in vitro*. In contrast, *Dm*pol γ has a heterodimeric composition, with one catalytic and one accessory subunit (αβ) [34]; both folding and stability are interdependent, as evidenced by the fact that in a heterologous system, the two subunits must be co-expressed to reconstitute the *Dm*pol γ holoenzyme [35]. Furthermore, subunit interactions in the insect enzyme occur at multiple sites along the polypeptides [36]. That the differences in pol γ stimulation by the fly and human mtSSBs results from differences in pol γ structure is supported by the fact that both *Dm*mtSSB and *Ec*SSB stimulate similarly *Dm*pol γ [37]. Additional support for this hypothesis is also provided by a recent report, which shows that each protomer of the dimeric human accessory subunit serves distinct roles in DNA synthesis by *Hs*pol γ [38]. Thus, we speculate that the function of the *Hs*pol γ-β dimer in the human mtDNA replisome is at least in part performed by *Dm*mtSSB in the *Drosophila* system. Further investigation of the mechanism of pol γ stimulation by mtSSB is clearly warranted to promote understanding of the species-specific roles of these proteins at the mtDNA replication fork.

Our analysis of stimulation of *Hs*pol γ by the deletion variants of *Hs*mtSSB revealed an interesting and surprising feature: the lack of the N- and/or C-terminus of *Hs*mtSSB increases its capacity to stimulate *Hs*pol γ under low ionic strength conditions. This suggests that both termini may actually modulate the DNA polymerase activity of *Hs*pol γ by inhibiting its stimulation by *Hs*mtSSBwt. Whether this modulation is mediated through physical or functional interactions only remains to be determined, but it is clear that the relevant interactions do not involve a positive regulation, as is the case for *Ec*SSB, T7 gp2.5 and T4 gp32, and their respective DNA polymerase partners at the replication fork. In considering the electrostatic forces that may govern functional interactions, we examined the predicted isoelectric points (pIs) for various structural elements in *Hs*pol γ-α and wild-type *Hs*mtSSB. We were especially interested in a fragment of the spacer region domain of *Hs*pol γ-α assigned as the intrinsic processivity (IP) sub-domain in the recent crystal structure [33], because earlier studies from our lab suggested that residues in this sub-domain are important for the functional interaction between *Drosophila* pol γ and its cognate mtSSB [39]. Consistent with the hypothesis that the functional interactions between *Hs*pol γ and *Hs*mtSSB are electrostatic, we found a pI of 8.2 for wild-type *Hs*mtSSB, and 5.7 for the IP sub-domain of *Hs*pol γ-α, suggesting that increasing salt in the pol assays disrupts the electrostatic forces that allow *Hs*pol γ to displace *Hs*mtSSB from ssDNA. The only other domain of *Hs*pol γ-α that carries an overall negative charge is the accessory-interacting domain (pI 4.7), which interacts tightly with the proximal protomer of the pol γ-β dimer. In examining the terminal deletion variants of *Hs*mtSSB we found pIs of 9.0 (ΔC), 9.2 (ΔN) and 9.5 (ΔNΔC), suggesting that lack of either or both termini causes a significant increase in the overall positive charges of the protein that may strengthen its interactions with *Hs*pol γ, stimulating DNA synthesis and allowing *Hs*pol γ to displace it from the ssDNA. At the same time, the ssDNA-binding affinities of the variant *Hs*mtSSBs are unaffected over the range of 20–100 mM KCl (Fig. 4 and data not shown). As the *Hs*mtSSB becomes more positively charged without its termini, the electrostatic forces between *Hs*pol γ and *Hs*mtSSB likely increase at 20 mM KCl, increasing the stimulation and the ability of *Hs*pol γ to displace the *Hs*mtSSB variants more easily. However, whereas ssDNA binding by *Hs*mtSSB is stable at 50 mM KCl, this salt concentration is apparently sufficient to disturb interactions between *Hs*pol γ and both the wild-type and variant forms of *Hs*mtSSB, giving rise to its inhibitory effects. Although the effects we observe are modest, it seems possible that given the fluctuating ionic conditions that occur in the mitochondrion [40], [41], they may play a role in regulating the interactions between *Hs*pol γ and *Hs*mtSSB *in vivo*.

In contrast with *Hs*pol γ, the stimulation of *Hs*mtDNA helicase by *Hs*mtSSB proteins is not salt-dependent, and the deletion variants of *Hs*mtSSB do not show a higher stimulatory effect than *Hs*mtSSBwt on the DNA unwinding activity of *Hs*mtDNA helicase. Taking into consideration that *Ec*SSB may not stimulate *Hs*mtDNA helicase as well as its cognate SSB [9], this result argues that the N- and C-terminal regions of *Hs*mtSSB are not involved directly in the 8-fold stimulation of *Hs*mtDNA helicase. However, it has been shown that *Ec*SSB can replace T7 gp2.5 *in vitro* to stimulate the T7 DNA polymerase holoenzyme activity on a singly-primed ssDNA template and in strand-displacement assays (the latter also involves the function of T7 gp4 primase-helicase), but it fails to promote either coupled leading and lagging strand synthesis *in vitro*, or the growth of bacteriophage T7 mutants lacking the gp 2.5 gene, both of which require the coordinated function of the T7 replisome [42], [43]. Therefore, our assays may be limited in assessing biochemically the possible defects of *Hs*mtSSB variants. In any case, one might argue that the mtDNA replication fork most likely comprises other unidentified components in addition to pol γ, mtDNA helicase and mtSSB, especially given the complexity of the myriad processes that occur in the mitochondrion [44], and the various modes of mtDNA replication that operate *in vivo* [45], [46], [47], which ensure appropriate mtDNA copy number and mitochondrial gene expression. Thus, physiological analysis of these and other mtSSB mutants, in addition to development of new *in vitro* assays that reconstitute fully the mtDNA replisome, will be informative in understanding the mechanism of mtDNA replication.

## Materials and Methods

### Nucleotides and nucleic acids

Unlabeled deoxy- and ribonucleotides were purchased from Amersham Bioscience. [α-^32^P]dATP and [γ-^32^P]ATP were purchased from MP Biomedicals. Recombinant M13 (10,650 nt) and pBSKS+ (2,958 nt) DNAs were prepared by standard laboratory methods. Oligodeoxynucleotides complementary to these DNAs were synthesized in an Applied Biosystems oligonucleotide synthesizer. The singly-primed M13 DNA used in DNA polymerase assays was prepared as described previously [13]. For the DNA unwinding assays, a 60-mer oligodeoxyribonucleotide (5′T_(40)_AGGTCGTTCGCTCCAAGCT3′) was radiolabeled at its 5′-end. The kinase reaction (50 µL) contained 50 mM Tris-HCl, pH 8.3, 10 mM MgCl_2_, 0.1 mM EDTA, 5 mM dithiothreitol (DTT), 0.1 mM spermidine, [γ-^32^P]ATP (0.66 µM, 4500 Ci/mmol), 700 pmol (as nt) of oligonucleotide, and 20 units of T4 polynucleotide kinase (New England BioLabs). Incubation was for 30 min at 37°C, and the 5′-end-labeled 60-mer oligonucleotide was purified using a Micro Bio-Spin P-30 Tris chromatography column (Bio-Rad), and annealed to pBSKS+ single-stranded plasmid DNA at 65°C for 60 min, followed by incubation at 37°C for 30 min, to generate a 20 bp double-stranded region with a 40-nt 5′-tail (the DNA unwinding substrate). The 48-mer oligodeoxynucleotide (5′GGACTATTTATTAAATATATTTAAGAACTAATTCCAGCTGAGCGCCGG3′) used in gel mobility shift assays was radiolabeled at its 5′-end as described above.

### Mutagenesis and purification of *Hs*mtSSB proteins

The *Hs*mtSSB deletion variants were constructed by cloning of PCR fragments containing the coding region for *Hs*mtSSBΔN, ΔC and ΔNΔC into the *Nde*I site of the pET11a vector. PCRs were performed using the coding region of the mature *Hs*mtSSBwt cloned in pET11a vector as template, *Pfu* DNA polymerase (Stratagene) and standard laboratory methods. The oligonucleotides used for PCR mutagenesis were: 5′CCCGGGCATatgCTTGAAAGATCCCTGAATCG3′ and 5′CCCGGGCATATGCTACTCCTTCTCTTTCGTCTGG3′ for *Hs*mtSSBΔN; 5′CCCGGGCATATGGAGTCCGAAACAACTACCAG3′ and 5′CCCGGGCATATGctaACTCAGAAATATAATATTATCAG3′ for *Hs*mtSSBΔC; and 5′CCCGGGCATatgCTTGAAAGATCCCTGAATCG3′ and 5′CCCGGGCATATGctaACTCAGAAATATAATATTATCAG3′ for *Hs*mtSSBΔNΔC. The underlined sequences correspond to *Nde*I restriction sites, and the lower case letters indicate the sites where mutations were introduced into the *Hs*mtSSB cDNA to create new start and stop codons. BL21(DE3) cells containing pET-11a plasmid expressing *Hs*mtSSBwt and deletion variants were grown at 37°C with aeration in L-broth containing 0.1 mg/mL of ampicillin. When the bacterial cell culture reached an optical density of 0.6 at 595 nm, isopropyl β-D-1-thiogalactopyranoside was added to 0.2 mM, and the culture was incubated further for 3 hr. Cells were harvested by centrifugation, washed in 50 mM Tris-HCl, pH 7.5, 10% sucrose (Tris-sucrose), frozen in liquid nitrogen, and stored at -80°C. All further steps were performed at 0–4°C, and all buffers contained 5 mM DTT, 1 mM phenylmethylsulfonyl fluoride, 10 mM sodium metabisulfite, and 2 ug/mL leupeptin. The cell pellet was thawed on ice, and cells were resuspended in 1/25 volume of original cell culture in Tris-sucrose and lysed by addition of 5 X lysis buffer (1 M NaCl, 10 mM EDTA, and 10% sodium cholate – for *Hs*mtSSBΔC purification, 7.5% n-dodecyl-β-D-maltoside instead of 10% sodium cholate) to a final 1 X concentration, followed by incubation for 30 min on ice and freezing in liquid nitrogen. After thawing on ice, the suspension was centrifuged at 17500×*g* for 30 min. The supernatant (soluble Fr I) was loaded onto a Blue Sepharose column equilibrated with 10 column volumes (CV) of 35 mM Tris-HCl, pH 7.5, 10% glycerol, 2 mM EDTA, 0.2 M NaCl at a packing ratio of 5–7 mg of total protein per mL of resin. The column was washed with 1 CV of equilibration buffer and 3 CV of 35 mM Tris-HCl, pH 7.5, 10% glycerol, 2 mM EDTA, 0.25 M NaSCN. The bound protein was eluted with 8 CV of a 0.4–1.2 M NaSCN linear gradient, followed by a final elution step of 1.5 M NaSCN buffer (2 CV). Fractions containing *Hs*mtSSB were pooled (Fr II) and dialysed against buffer containing 60 mM KPO_4_, pH 7.6, 10% glycerol, 2 mM EDTA (Fr IIb) (for *Hs*mtSSBΔC, dialysis buffer contained 40 mM KPO_4_, pH 7.6). Fr IIb was then loaded onto a phosphocellulose column equilibrated with dialysis buffer at a packing ratio of 0.5 mg of total protein per mL of resin. The column was washed with 2.5 CV of the same buffer and the proteins were eluted with 5 CV of a 60–150 mM KPO_4_ linear gradient, followed by a final step of 350 mM KPO_4_ buffer (2 CV). The *Hs*mtSSB proteins typically elute at ∼80 mM KPO_4_. Pooled fractions (Fr III) were concentrated to ∼1 mg/mL of protein in a Centricon-30 spin concentrator (Amicon) treated with 5% Tween 20 (Fr IIIb). Fr IIIb was frozen in liquid nitrogen and stored at −80°C.

### Purification of *Hs*pol γ and *Hs*mtDNA helicase

Recombinant human pol γ-α exo^–^ and pol γ-β were prepared from *Sf*9 and bacterial cells, respectively, as described by Oliveira and Kaguni [15]. Recombinant human mtDNA helicase was prepared from *Sf*9 cells, as described by Ziebarth *et al.* [48].

### Glycerol gradient sedimentation and gel filtration

*Hs*mtSSBwt and variants (100 µg) were layered onto preformed 12–30% glycerol gradients (10 mL) containing 35 mM Tris-HCl, pH 7.5, 100 mM NaCl, 2 mM EDTA, 5 mM DTT, 1 mM phenylmethylsulfonyl fluoride, 10 mM sodium metabisulfite, and 2 ug/mL leupeptin. Centrifugation was at 264,000×*g* for 63 hrs at 4°C in a Beckman SW41 rotor. Fractions were analyzed by SDS-PAGE and Coomassie blue staining. For Superdex 75 gel filtration, 200 µg *Hs*mtSSBwt and variants were chromatographed on a column equilibrated with the buffer described above containing 8% glycerol at a flow rate of 0.25 mL/min at 4°C. Fractions were analyzed by SDS-PAGE and Coomassie blue staining to confirm UV trace recordings. Standard protein markers used in both procedures were: bovine serum albumine (BSA, 4.85 *S*, *R_S_*  = 3.61 nm), carbonic anhydrase (CA, 3.23 *S*, *R_S_*  = 2.43 nm) and lysozyme (LYS, 1.91 *S*, *R_S_*  = 1.5 nm). The data were plotted as *S* value *versus* fraction number to obtain a sedimentation coefficient, and as *R_S_* value *versus* the peak of protein elution in mL to obtain the Stokes radii for the *Hs*mtSSB proteins. The native molecular mass of the proteins were calculated using the formula: MW  = 3.909 x *S* value x *R_S_*.

### ssDNA binding and gel mobility shift assay

Reaction mixtures (20 µL) contained 20 mM Tris-HCl, pH 7.5, 1 mM DTT, 4 mM MgCl_2_, 50 mM NaCl, 36 fmol 5′-end-labeled 48-mer, and the indicated amounts of the *Hs*mtSSB proteins. Incubation was at 20°C for 10 min. Samples were processed and electrophoresed in 6% native polyacrylamide gels. The amounts of shifted and free oligonucleotide were quantitated as follows: % ssDNA bound  =  (V_S_/(V_S_+V_F_)) ×100, where V_S_ represents the volume of the shifted and V_F_ the volume of unshifted oligonucleotide in the sample lane of interest.

### DNA polymerase γ stimulation assays

Reaction mixtures (50 µL) contained 50 mM Tris-HCl, pH 8.5, 4 mM MgCl_2_, 400 µg/ml bovine serum albumin, 10 mM DTT, 20–100 mM KCl, 20 µM each dGTP, dATP, dCTP and dTTP, [α-^32^P]dATP (2 µCi), 10 µM (as nt) singly-primed recombinant M13 DNA, 10 ng *Hs*pol γ-α exo^−^ Fr IV, 48 ng *Hs*pol γ-β Fr III, and the indicated amounts of *Hs*mtSSB proteins. Incubation was at 37°C for 30 min. Samples were processed and nucleotide incorporation was quantitated in a liquid scintillation counter.

### DNA unwinding assays

Reaction mixtures (50 µL) contained 20 mM Tris-HCl, pH 7.5, 10% glycerol, 500 µg/mL bovine serum albumin, 10 mM DTT, 4 mM MgCl_2_, 3 mM ATP, 50 mM KCl (unless stated otherwise), 0.4 nM of DNA unwinding substrate, 3.5 nM of mtDNA helicase (as hexamer), and the indicated concentrations of *Hs*mtSSB proteins. The reactions were pre-incubated at 37°C for 10 min prior to the addition of the helicase. Once the helicase was added, the reactions were incubated further at 37°C for 30 min and then stopped by the addition of 5 µL of 10 X stop solution (6% SDS, 100 mM EDTA, pH 8.0), followed by 5 µL of 10 X loading buffer (50% glycerol, 0.25% bromophenol blue). DNA products were fractionated from substrate by electrophoresis in a 22% polyacrylamide gel (59∶1 acrylamide/bisacrylamide) using 1 X TBE (90 mM Tris-HCl-borate, 2 mM EDTA) at 600 V for ∼30 min. After electrophoresis, the gel was dried under vacuum with heat, and exposed to a Phosphor Screen (Amersham Biosciences). The data were analyzed by scanning the Phosphor Screen using a Storm 820 Scanner (Amersham Biosciences), and the volume of each band were determined, and background subtracted, by computer integration analysis using ImageQuant version 5.2 software (Amersham Biosciences). For all reactions, DNA unwinding is defined as the fraction of radiolabeled DNA species that is single-stranded (product), as follows: % unwinding  =  (V_P_/(V_S_ + V_P_)) ×100, where V_P_ represents the volume of the product and V_S_ the volume of unreacted substrate in the sample lane of interest.
